# Outcomes of Patients with Early Onset Colorectal Cancer Treated in a UK Specialist Cancer Center

**DOI:** 10.3390/cancers11101558

**Published:** 2019-10-14

**Authors:** Alexandros Georgiou, Shelize Khakoo, Penelope Edwards, Anna Minchom, Kyriakos Kouvelakis, Eleftheria Kalaitzaki, Natalie Nobar, Vanessa Calamai, Maria Ifijen, Olga Husson, David Watkins, Sheela Rao, Ian Chau, David Cunningham, Naureen Starling

**Affiliations:** 1 Department of Medicine, The Royal Marsden Foundation Trust, London and Surrey SM2 5PT, UK; a.georgiou@nhs.net (A.G.); shelize.khakoo@rmh.nhs.uk (S.K.); penelope.edwards@rmh.nhs.uk (P.E.); anna.minchom@icr.ac.uk (A.M.); kyriakos_kouvelakis@yahoo.gr (K.K.); eleftheria.kalaitzaki@rmh.nhs.uk (E.K.); natalie.nobar@gmail.com (N.N.); vanessacalamai@alice.it (V.C.); maria.ifijen@rmh.nhs.uk (M.I.); olga.husson@icr.ac.uk (O.H.); david.watkins@rmh.nhs.uk (D.W.); sheela.rao@rmh.nhs.uk (S.R.); ian.chau@rmh.nhs.uk (I.C.); david.cunningham@rmh.nhs.uk (D.C.); 2 Department of Clinical Studies, Institute of Cancer Research, London SM2 5NG, UK

**Keywords:** early onset, young adults, colorectal cancer, chemotherapy, treatment, outcomes

## Abstract

The incidence of early onset colorectal cancer (EOCRC) is rapidly increasing, but there remains paucity of outcome data for young CRC patients. We reviewed the characteristics and outcomes of 241 adults, age <50, who were diagnosed with EOCRC between January 2009 and December 2014. Median age was 42, 56% were male, and 7% had hereditary etiology. Seventy percent had left-sided primaries. At diagnosis, 11%, 50%, and 39% had stage II, III, and IV CRC. Of the patients with stage II and III CRC who underwent curative surgery, 60% and 88% had adjuvant chemotherapy, with 5-year relapse free survival of 82% and 74% respectively. Of the 123 patients with metastatic (m) EOCRC, 93%, 63%, 33%, and 12% had 1st, 2nd, 3rd, and 4th line systemic anticancer therapy (SACT) respectively. For first line SACT, 99% had doublet chemotherapy, with bevacizumab or an anti-EGFR antibody in 57%. Median overall survival (mOS) of mEOCRC patients was 20.1 months (95% C.I: 15.9–23.2). Younger age and signet cells were associated with shorter mOS, whereas more lines of SACT and curative metastasectomy with longer mOS. Metastatic EOCRC patients had poorer outcomes than expected, despite optimal multimodality treatment. This suggests an aggressive disease biology that warrants further research and therapy development.

## 1. Introduction

Colorectal cancer (CRC) is the third most common cancer diagnosis worldwide [1]. It is traditionally considered a disease of older adults, therefore, in the United Kingdom (UK) fecal occult blood CRC screening is typically reserved for people over the age of 60. Similarly, CRC screening is limited to patients older than 50 years in the United States of America (USA) [2]. Early onset colorectal cancer (EOCRC) is defined as diagnosis of CRC in patients younger than 50 years old. Over recent years, there has been a significant increase in the incidence of EOCRC, with the highest rate of increase seen in rectal cancer. Epidemiological studies across the globe, including in Europe, Canada, USA, Australia, China, India, Japan, and Brazil, report a similar rise in incidence of CRC in young adults over the last 20–25 years [3,4,5,6]. In the USA, colon cancer incidence rates increased by 1.0% to 2.4% annually since the mid-1980s in adults age 20 to 39 years and by 0.5% to 1.3% since the mid-1990s in adults aged 40 to 54 years. Rectal cancer incidence rates have been increasing for longer and more rapidly (3.2% annually from 1974 to 2013 in adults aged 20–29 years) [7]. It is estimated that around 11% of colon cancers and 18% of rectal cancers occur in individuals younger than 50 years of age [8].

EOCRC appears to be a heterogeneous disease with distinct clinicopathological and molecular characteristics. Young adults tend to have a higher prevalence of low-grade tumor differentiation, mucinous and signet ring cell tumors when compared to older patients [9,10]. A proportion of EOCRC can be attributed to hereditary syndromes. It is estimated that up to 20–30% of EOCRC has a hereditary component. Of these, only 3–5% have a well-characterized genetic basis [11]. The rate of EOCRC attributed to known hereditary syndromes is heavily influenced by the age groups analyzed, with patients <35 years old exhibiting the highest rates [12]. Lynch Syndrome (LS) is the most prevalent hereditary syndrome, followed by familial adenomatous polyposis (FAP), Peutz–Jeghers syndrome, juvenile polyposis syndrome, and others. The reported prevalence of LS in EOCRC cohorts is variable ranging from 4% to 20% [13]. The underlying pathogenesis of LS relates to the presence of germ-line mutations in one of the DNA mismatch repair (MMR) genes, which in itself results in microsatellite instability (MSI) [14]. In addition to MSI secondary to LS, MSI tumors can also be found in sporadic, nonhereditary cases. The majority of these cases occur secondary to epigenetic silencing of the *MLH1* gene. Overall it is estimated that 15% to 20% of cases of EOCRC have MSI, a rate that is higher than that of older adults, albeit still a minority group [15].

The current literature relating to EOCRC is mainly limited to epidemiological studies that characterized the incidence and prognosis of younger adults with CRC. There is currently very sparse literature describing treatment pathways of these patients and their associated outcomes.

We aimed to profile the clinicopathological characteristics and treatment outcomes of patients with EOCRC (age <50 years) in the real-world setting of a single high-volume tertiary UK cancer center.

## 2. Results

### 2.1. Patient Characteristics

During the 6-year period of 1 January, 2009 to 31 December, 2014, a total of 561 patients aged 18–49 were registered at Royal Marsden Hospital (RMH) and had a diagnosis of CRC. Out of the 561 patients, 241 (43%) patients met our inclusion criteria. The excluded patients had received their treatment in other UK centers or overseas and therefore their treatment outcomes were not available for extraction. The majority of excluded patients attended RMH for a single specialist review with the surgical, clinical, or medical oncology teams, including consultations to discuss participation in clinical trials.

The median age of all included patients was 42 years old (range 19 to 49) and 56% were male (Table 1). In total, 103 (43%) patients were <40 years old. The majority of patients declared a white British (65%) ethnicity followed by Asian or Asian British (10%). The Body Mass Index (BMI) within 3 months of diagnosis was available for 227 (94%) patients. The median BMI was 24.9 kg/m^2^ (95% C.I 24.3–25.2), with a mean of 25.1 kg/m^2^ (range 16.4–40.9). Overall, 231 (96%) patients had an Eastern Cooperative Oncology Group (ECOG) performance status (PS) of 0 or one at diagnosis.

Twenty-seven (11%) patients presented with stage II disease. Most patients presented with stage III disease (*n* = 120, (50%)) and 93 (39%) patients had metastatic disease at diagnosis.

Overall the majority of patients had a left sided primary tumor (70%) with rectum/rectosigmoid being the most prevalent primary site (*n* = 102 (43%)) followed by descending or sigmoid colon (*n* = 64 (27%)) and caecum/ascending colon (*n* = 57 (24%)).

There were 23 cases (10%) of mucinous adenocarcinoma. Six (2%) patients had signet cells and out of these, five (83%) had metastatic disease at diagnosis. Most primary tumors (73%) were moderately differentiated, whereas 19% were poorly differentiated.

MMR status, assessed by immunohistochemistry, was available in 165 (68%) patients, and of these, 148 (90%) had MMR proficient tumors. In patients with known MMR status with stage II disease at diagnosis, 12% had MMR deficient tumors. In patients with stage III disease the rate of MMR deficiency was 16%, whereas in patients with stage IV disease at diagnosis, only one (2%) patient had MMR deficient CRC. Out of the 17 patients that were known to have MMR deficient tumors, 11 (65%) patients were known to have Lynch syndrome.

A total of sixteen patients (7%) with EOCRC were found to have a known hereditary syndrome. As expected, the most prevalent hereditary syndrome was Lynch syndrome (*n* = 11).

Routine molecular characterization of patients’ tumors with stage IV disease evolved during the course of the 6-year period included (January 2009–December 2014). In 2009, molecular characterization was not part of patients’ routine care. Subsequently, *KRAS* mutation testing was carried out and by 2014, exon 2–4 *NRAS/KRAS* and *BRAF* mutation testing was considered standard of care. As a result of this, the majority of the included patients have an unknown *NRAS* and *BRAF* genetic mutation status. Out of the 134 patients with known *KRAS* status, 37% had a known activating mutation.

### 2.2. Treatment and Outcomes in Patients with Stage II and III EOCRC

#### 2.2.1. Neo-Adjuvant Chemoradiation

A total of 48 (80%) patients with stage II or III rectal cancer were treated with neo-adjuvant radiotherapy and out of these patients, 44 (92%) received long course chemo-radiation with concomitant capecitabine or 5FU. On pretreatment MRI imaging, a total of 23 (48%) patients had documented circumferential resection margin involvement (CRM) and 32 (67%) patients had evidence of extramural vascular invasion (EMVI). A total of four (8%) patients had evidence of local or systemic relapse after completion of radiotherapy (+/− chemotherapy).

#### 2.2.2. Curative Surgery and Adjuvant Chemotherapy

**Figure 1:**
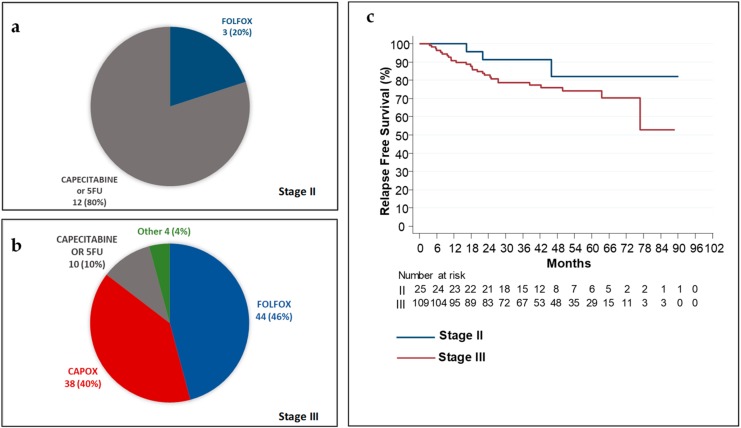
Adjuvant chemotherapy and Relapse Free Survival in patients with stage II and II colorectal cancer (CRC). (**a**) Adjuvant chemotherapy regimens in 15 patients with stage II CRC; (**b**) Adjuvant chemotherapy regimens in 96 patients with stage III CRC; (**c**) Kaplan–Meier estimates for relapse-free survival (RFS) in all patients with Stage II and III disease. 5FU: 12 cycles, each 14-day cycle; 5FU bolus 400 mg/m^2^, 5FU 2400 mg/m^2^ infusion over 48 h and folinic acid 350 mg. Capecitabine 8 cycles each 21-day cycle; capecitabine 2000 mg/m^2^ per day in two divided doses for 14 days followed by 7-day rest. CAPOX: 8 cycles, each 21-day cycle; capecitabine 1700 mg/m^2^ per day in two divided doses for 14 days followed by 7-day rest and oxaliplatin 130 mg/m^2^ on day 1. FOLFOX: 12 cycles, each 14-day cycle; 5FU bolus 400 mg/m^2^, 5FU 2400 mg/m^2^ infusion over 48, folinic acid 350 mg and oxaliplatin 85 mg/m^2^ on day 1. Other: experimental combinations that were administered only within clinical trials: experimental regimens were FOLFOX or CAPOX with targeted therapies.

A total of 15 (60%) out of 25 patients with stage II disease that had a documented history of curative surgery went on to have adjuvant chemotherapy. Of these patients 80% had adjuvant capecitabine or 5FU alone. The 5-year relapse-free survival (RFS) for stage II patients was 82.2% (Figure 1).

Out of the 109 patients with stage III disease that had curative surgery, 96 (88%) patients were treated with adjuvant chemotherapy. Out of the patients that received adjuvant chemotherapy, 86% patients had standard of care oxaliplatin-based doublet chemotherapy, while four patients had oxaliplatin-based doublet in combination with targeted therapies within clinical trials. The 5-year relapse-free survival (RFS) for stage III patients was 74.1% (Figure 1).

Out of all the patients that were treated with adjuvant chemotherapy, 13 (12%) patients had discontinuation of their adjuvant chemotherapy earlier than planned, while 30% required a treatment delay during the 6-month course of their adjuvant chemotherapy. Moreover, in 34 of the 88 (39%) patients that were treated with oxaliplatin-based doublet chemotherapy, oxaliplatin was discontinued early, during their 6-month course of treatment.

### 2.3. Treatment and Outcomes in Patients with Stage IV EOCRC

A total of 123 patients with mEOCRC were included. Out of these, 93 (76%) patients had stage IV disease at diagnosis, while two (2%) patients had stage II and 28 (23%) had stage III disease at diagnosis and relapsed with metastatic disease subsequently. A total of 114 (93%) patients were treated with SACT for mEOCRC, while the rest of the patients were either of a poor PS at diagnosis and did not receive SACT or had oligometastatic disease and were treated with metastasectomy alone.

**Figure 2:**
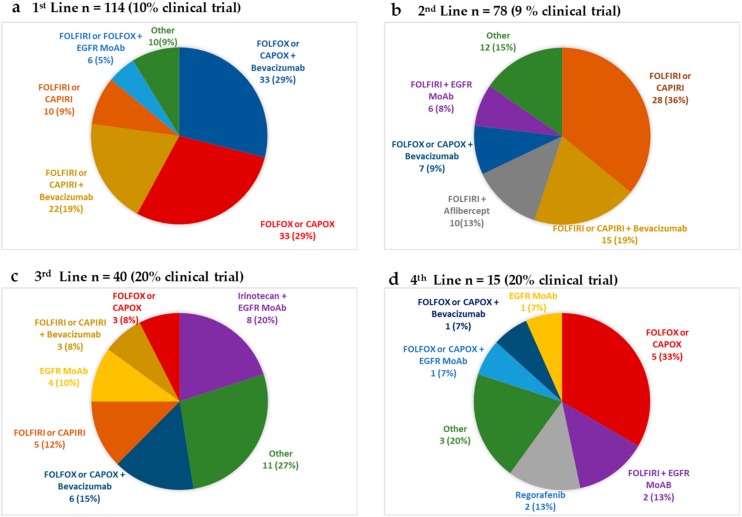
The most frequently used systemic anticancer therapy (SACT) regimens in the metastatic setting in (**a**) first line, (**b**) second line, (**c**) third line, and (**d**) fourth line setting. Epidermal Growth Factor Receptor (EGFR) Monoclonal antibody (MoAb) therapy (cetuximab 500 mg/m^2^ 2-weekly or panitumumab 6 mg/kg 2-weekly) was only offered in patients with known *KRAS* wild type tumors. FOLFIRI (14 day cycle: 5FU bolus 400 mg/m^2^ and 5FU 2400 mg/m^2^ infusion over 48 h, folinic acid 350 mg and irinotecan 180 mg/m^2^ day 1), CAPIRI (21 day cycle: capecitabine 1700 mg/m^2^ per day in two divided doses for 14 days followed by 7 day rest and irinotecan 200 mg/m^2^ on day 1), FOLFOX (14 day cycle: 5FU bolus 400 mg/m^2^,5FU 2400 mg/m^2^ infusion over 48 h folinic acid 350 mg and oxaliplatin 85 mg/m^2^ on day 1), CAPOX (21 day cycle: capecitabine 1700 mg/m^2^ per day in two divided doses for 14 days followed by 7 day rest and oxaliplatin 130 mg/m^2^ on day 1). Bevacizumab in all 3-weekly regimens at 7.5 mg/kg, 2 weekly regimens at 5 mg/kg. Aflibercept at 4 mg/kg 2 weekly. Regorafenib 4 weekly cycles at 160 mg once a day (OD) for 21 days followed by 7-day rest.

The most commonly administered regimens per line of SACT are shown in Figure 2. In the first line metastatic setting, out of the 114 patients that received SACT, 113 (99%) patients received a doublet chemotherapy regimen, with the addition of bevacizumab in 48% of patients.

The documented partial response (PR) stable disease (SD) and progressive disease (PD) rates in the first line setting regardless of regimen were 40%, 23%, and 32% respectively. The median PFS in the first line setting, was 6.9 (95% C.I: 5.8–9.4) months (Table 2).

Of the patients that had first line SACT, 78 (68%) went on to have a 2nd line SACT. The most commonly used regimen in the second line setting was irinotecan-based combination with or without the addition of bevacizumab (36% and 19% respectively). The documented PR, SD, and PD rates were 17%, 26%, and 51% respectively. The median progression-free survival (PFS), irrespective of regimen, in the second line setting was 5.0 (95% C.I: 3.4–6.1) months (Table 2).

A total of 33%, 12%, and 5% of all patients with mEOCRC went on to receive 3rd, 4th, and 5th line treatments respectively. The most commonly utilized SACT regimens in the 3rd and 4th line setting are shown in Figure 2 and outcomes per line of therapy in Table 2. As the number of lines of SACT increased, so did the participation of treatment within a clinical trial setting, with 20% of patients treated within clinical trials from the third line setting onwards.

The median overall survival (mOS) increased with increasing number of sequential lines of SACT (Table 2). Patients that were only able to receive a total of one line of SACT had a mOS of 9.0 (95% C.I: 4.1–27.7) months. Patients that received a total of two lines had a mOS of 14.9 (95% C.I: 9.6–19.0) months, while those that had a total of three and four lines of SACT had a mOS of 18.7 (95% C.I: 14.3–28.6) and 31.7 (95% C.I: 20.2–46.5) months respectively.

A total of 17 (14%) patients underwent metastasectomy with curative intent. In addition, patients with metastatic disease received the following palliative treatments during the course of their treatment: 27 (22%) patients had palliative radiotherapy, 21 (17%) patients had palliative surgery to the primary tumor, 11 (9%) patients had radiofrequency ablation (RFA) to the liver and or lung metastases, four (3%) patients had stereotactic radiosurgery, four (3%) patients had selective internal radiation therapy (SIRT) to liver metastasis, and two (2%) patients had microwave ablation to the liver.

The mOS for all patients with mEOCRC was 20.1 months (95% C.I: 15.9–23.2). For the 93 patients that presented with stage IV disease (*n* = 93), mOS was 18.8 months (95% C.I: 14.3–22.6). In the case of patients that relapsed with systemic metastases following an initial diagnosis of stage II (*n* = 2) and III (*n* = 28) disease, the mOS from the time of diagnosis of metastatic disease was 9.1 (95% C.I N/A) months and 26.2 (95% C.I: 15.7–49.6) months respectively.

### 2.4. Prognostic Markers of Survival in Patients with Stage IV CRC

**Figure 3:**
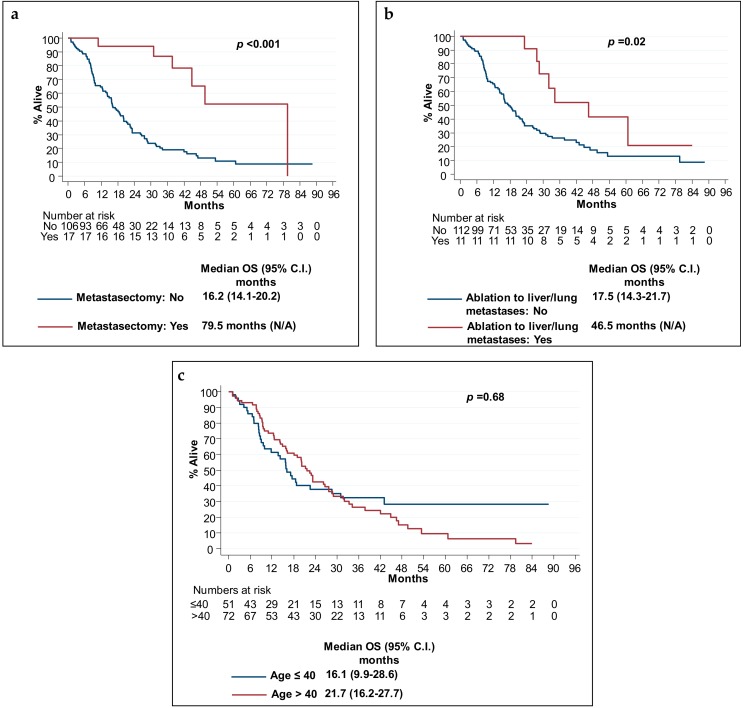
Prognostic markers for overall survival in metastatic disease. Kaplan–Meier estimates of overall survival according to (**a**) whether patients underwent metastasectomy with curative intent, (**b**) whether they had radiofrequency ablation to liver and/or lung metastases, and (**c**) age at diagnosis, ≤40 years old or >40 years old. *p*-values refer to log rank test.

As expected, the 17 (14%) patients that had curative metastasectomy had a statistically significant longer mOS (*p* < 0.001), when compared to patients that did not (Figure 3). In addition, 11 (9%) patients that had metastatic disease that was deemed treatable with local ablative therapy to the liver and/or lung metastases also had significantly longer mOS (Figure 3). The latter analysis may have been confounded by the fact that these patients were likely to have had oligo-metastatic disease.

Patients with signet cells (5%) had a significantly shorter mOS, although this analysis was limited by the small number of patients with signet cell histology (Table 3).

Younger age at presentation was associated with a numerically shorter mOS. As shown in Figure 3c, patients age ≤40 had mOS of 16.1 (95% C.I 9.9–28.6) compared to 21.7 months in patients age >40 group (95% C.I 16.2–27.7).

When considering age groups per decade cohorts there was an inverse relationship between age and mOS, with youngest patients having numerically the shortest mOS (*p* < 0.05):

- age 20–29 (*n* = 13), mOS 15.9 (95% C.I 7.0–43.0)
- age 30–39 (*n* = 37), mOS 17.1 (95% C.I not reached)
- age 40–49 (*n* = 72), mOS 21.7 (95% C.I 16.2–27.7)

The location of primary tumor (left vs. right sided) and *KRAS* and *BRAF* mutation status did not significantly impact on mOS (Table 3). The *BRAF* analysis was limited by the small number of patients that had known *BRAF* genotyping results, although, as expected, patients with *BRAF* wild type tumors had a numerically longer mOS when compared to patients with *BRAF* mutant tumors (Table 3).

## 3. Discussion

It is now recognized that the incidence of EOCRC is increasing rapidly at a global scale [3,4,5,6]. In the USA it is predicted that based on current trends, in 2030, the incidence rates for colon and rectal cancers will increase by 90.0% and 124.2%, respectively, for patients aged 20 to 34 years and by 27.7% and 46.0%, respectively, for patients aged 35 to 49 years [16].

In our study, a total of 561 patients, age <50, with EOCRC were registered over the 6-year period from January 2009 to December 2014. The sheer number of patients with EOCRC that were registered at a single cancer center, further highlights that patients with EOCRC represent a sizeable group, who merit further consideration in terms of bespoke therapy development and configuration of services.

Out of the of 241 (43%) patients that met our inclusion criteria, 56% were male and the majority (65%) declared a White British ethnic background, followed by 10% that declared Asian/Asian-British background. Overall, the ethnic background data of our population, while reflective of the multi-cultural London population, also suggest that in the UK, EOCRC is likely to span all ethnic backgrounds. Studies to date sought to identify population subgroups with the highest predisposition to EOCRC. While there have been some discrepancies between the outcomes reported in different countries, overall, the increase in incidence rates of EOCRC appears similar between males and females [5]. In the USA, there is data to suggest that there are persistent disparities by race/ethnicity and possibly socio-economic status. However, these disparities may reflect the relative distribution of other risk factors that are known to be associated with CRC, such as obesity and type II diabetes in these subgroups [17,18,19]. Our cohort had a median BMI that was within the normal distribution, median of 24.9 kg/m^2^, albeit at the uppermost limit of normality.

In our population there was a left sidedness preponderance with 43% of patients having rectal or recto-sigmoid tumors as their primary site. Only 21% of patients presented with stage I or II disease, while 39% of patients had metastatic disease at diagnosis. Overall, our findings are in line with other studies that suggest that the incidence of rectal cancer is increasing most rapidly in young adults and that these patients tend to be diagnosed with advanced disease stage [19,20,21,22]. It is however worth noting that other epidemiological data from Europe suggests that the largest increase in CRC incidence in young adults is in fact of colon rather than rectal origin [3,23], thereby, challenging recent suggestions of screening strategies in young adults with flexible sigmoidoscopy alone [24].

Although we have not been able to investigate the prior diagnostic pathway of our patients, it has been previously suggested that young patients with CRC report symptoms for a longer period of time, prior to diagnosis, than their older counterparts [25,26]. It is possible that young CRC patients present with symptoms, such as bleeding per rectum, that are initially attributed to benign causes such as hemorrhoids, which may co-exist. On the other hand, young adults are more likely to have competing responsibilities and may be less likely to seek professional help early, as they may normalize or wrongly attribute their symptoms to something other than cancer, thereby further adding to potential diagnostic delay [27]. Globally, established CRC screening programs in their current shape include patients >50 years old and although there is no data to confirm this, it is possible that this may contribute to the fact that young patients tend to present with more advanced stage at diagnosis. Our data adds further evidence supporting recent publications that suggest that CRC screening should be more inclusive of younger patients [28]. Indeed, the American cancer society has recently recommended lowering the age to start CRC screening from 50 to 45 years with either a high-sensitivity stool-based test or a structural (visual) examination, depending on patient preference and test availability [29].

In our cohort a minority of patients had a known hereditary syndrome (7%), with the most prevalent syndrome being, as expected, Lynch syndrome. Overall the rate of known hereditary syndromes in our cohort is at the lower end of the spectrum when compared to previously reported studies [12,13]. This may have been due to the fact that other retrospective studies in EOCRC included a younger group by only including patients aged <40 years old. It is well established that the younger the median age of the EOCRC cohort, the higher the rate of hereditary etiology [12,19]. Patients with known hereditary syndromes remain an important proportion of the EOCRC population that require specialist cancer genetics input and specialized surveillance strategies. Nevertheless, it is also important to acknowledge that the majority of young adults with CRC are in fact patients with nonhereditary sporadic tumors.

Our molecular characterization results for MMR status as well as *KRAS/NRAS* and *BRAF* mutation rates reflect the fact that during the earlier years of data collection, molecular characterization was not routinely carried out, which is currently not the case. Our data is in line with the existing literature on rates of *KRAS* mutations in the general CRC population and also with that reported in other retrospective EOCRC series [30,31]. Our MMR deficiency rates are consistent with that reported by other studies that also suggested that MSI tumors are more common in EOCRC [15,31]. In line with the general CRC population, our EOCRC patients with MMR deficient tumors had an earlier stage at diagnosis when compared to patients with MMR proficient tumors. Only one in 17 (6%) patients with MMR deficient CRC presented with metastatic disease, as compared to our overall 39% rate of metastatic CRC at diagnosis. This is of clinical relevance, as patients with MMR deficient tumors are likely to respond to immune checkpoint inhibitors [32].

Importantly, to date, there are no prospective trials that were designed to explicitly investigate the efficacy of SACT in young CRC adults. As a consequence of this, the treatment of these patients is largely based on extrapolating results from clinical trials that enrolled older patients. We therefore sought to analyze the treatment patterns and outcomes of patients with EOCRC, who were treated within our UK specialist cancer center, as this would serve as a baseline for future prospective research.

In our patients with stage II disease, a higher than expected proportion (60%) went on to receive adjuvant chemotherapy. Clinical trials in age-unselected populations suggested that the absolute survival benefit of adjuvant chemotherapy in stage II CRC is in the order of 4% [33]. We speculate that the high uptake of adjuvant chemotherapy in our stage II EOCRC patients probably reflects the fact that younger patients are willing to have any treatment with possible benefit, even if the likelihood of benefit is low. Overall, the RFS rates for both stage II and III patients are comparable to that of historical clinical trial controls that enrolled all age groups [34]. It remains unknown as to whether intensification of adjuvant chemotherapy in EOCRC is of any benefit. Other retrospective trials also suggest that adults with EOCRC receive significantly more postoperative SACT at all stages, but they experience only minimal gain in adjusted survival compared with their older counterparts who receive less treatment [21,35]. A circulating tumor DNA (ctDNA)-based risk stratification strategy may permit for the better selection of patients that are likely to benefit from adjuvant chemotherapy and prospective trials investigating this are currently underway [36].

To our knowledge we are the first to profile the regimens used and outcomes per line of palliative SACT in young adults with mCRC. In the first line setting, 99% of our patients had doublet chemotherapy, with the addition of targeted therapy in more than half of the patients. The majority of patients went on to have standard of care second line SACT with a third of all metastatic patients having at least three lines of therapy. From the third line SACT setting onwards, 20% of patients had their treatment within clinical trials, while the remaining patients often had a rechallenge of previously used regimens. Many patients had other additional palliative treatments such as surgery, radiotherapy, and localized treatments such as RFA to the liver and/or lung metastases. These results are overall reflective of a fit and motivated cohort of patients, who were treated with all evidence-based multimodality therapies. Whilst noting the limitations associated with a single center (albeit a high-volume cancer specialist hospital) our data suggests that there may be scope for prospective clinical trials tailored specifically for young adults with CRC.

The partial response rate and median PFS rates in the first- and second-line metastatic setting compare unfavorably to published data in unselected patients in terms of age, including data from other real-world observational trials [30,37,38,39]. In our study, young patients that were able to have sequential SACT for metastatic disease had a numerically higher mOS, with a stepwise improvement in their mOS with increasing lines of SACT. It is however worth noting that during the data collection period included (January 2009–December 2014), first line triplet cytotoxic chemotherapy combination with FOLFOXIRI (a combination of 5FU, oxaliplatin, and Irinotecan) and bevacizumab was not considered a standard of care option, and therefore it was not one of the used regimens in our study [40]. Since 2014, there has been data in support of this regimen and we expect that it is now more commonly used in fit young adults with metastatic CRC, especially where a conversion approach is possible or in patients with known right sided or *BRAF* mutant primary tumors [41,42].

In the current literature there is scarcity of studies that reported the response to specific SACT. We could not identify any other trials that reported PFS and response rates (RR) per line of SACT in young patients with mCRC. An older trial, with a pooled analysis of patients from nine first line phase III chemotherapy trials using fluorouracil-based single-agent and combination chemotherapy, suggested that young age is modestly associated with poorer PFS but not OS or RR in treated patients with advanced CRC [43]. The only other trial that reported PFS rates in the first line SACT setting for young adults with mCRC by retrospectively assessing the effect of age on outcomes from 24 first line clinical trials, reported that younger and older age are associated with poorer OS and PFS when compared to middle-aged patients [44].

In our study, the mOS of 20.1 months (95% C.I: 15.9–23.2) for all patients with mEOCRC was lower than expected, particularly given the fact that these patients were otherwise of a good PS and were treated with what were at the time considered the most efficacious multimodality therapies available. One of the most notable findings of our study was the fact that younger age at diagnosis was in fact associated with a shorter mOS with an inverse relationship between age and mOS.

We identified signet cell histological subtype which was present in 5% of patients, as a poor prognostic marker for OS in mEOCRC. However, given the small number of patients with signet cell histology, this cannot explain the overall short mOS of this cohort or the shorter mOS amongst the youngest included patients. Previous retrospective studies compared the prognosis of patients with EOCRC to that of older counterparts. A recent review of these trials reported mixed results, with some studies indicating a poorer prognosis, whereas others support a comparable or even better prognosis in comparison with older patients [35]. In line with our results, there have been previous reports suggesting that the younger end of the EOCRC spectrum have inferior survival outcomes [31,44].

In the current literature there is preliminary evidence to suggest that the pathological and molecular characteristics of the tumors of young patients with EOCRC differ from that of older patients. In line with our results, signet cell histology is over-represented in young patients [9,11]. It is likely that the EOCRC is a heterogeneous cohort in terms of disease biology, with the youngest patients being likely to have the most aggressive disease phenotype, as also suggested by our results. While EOCRC can develop through MSI or chromosomal instability (CIN) pathways, most EOCRC exhibits MSS phenotypes. Genome-wide hypomethylation is a feature of a subgroup of EOCRC. In particular, a subset of EOCRC has hypomethylation of LINE-1 sequences compared to later onset CRC. LINE-1 hypomethylation has been associated with adverse clinical behaviors. Other groups have also reported an excess of tumors that are both MSS without CIN, also known as Microsatellite and Chromosome Stable (MACS) tumors, which tend to be over-represented in poor prognosis left sided EOCRC tumors [19,45]. A recent study that investigated the molecular characteristics, including classification as per the transcriptomic consensus molecular subtyping (CMS), also suggested that EOCRC is a heterogeneous cohort with distinct molecular characteristics. The youngest patients, age <30, had distinct signaling aberrations with a lower rate of MAPK pathway mutations and a higher rate of *CTNNB1* mutations. In patients younger than 40 years, CMS1 was the most common subtype, partly reflecting the higher rates of MSI. CMS3 and CMS4 were uncommon, whereas CMS2 was relatively stable across age groups [46]. Further research aiming to identify distinct molecular phenotypes of EOCRC is required as this may allow for future novel therapeutic interventions.

In addition to their differing needs in terms of anticancer therapies, it is also important to acknowledge that young patients with CRC are likely to be faced with other psychosocial concerns that are distinctly different to that of older patients. For example, these patients are likely to have concerns relating to the possible hereditary component of their disease as well as family planning and fertility preservation concerns, which should be addressed proactively during treatment planning. Other considerations include the impact of surgery, such as living with a stoma, as well as the impact of long-term side-effects of chemotherapy (such as oxaliplatin induced neuropathy) and late effect of radiotherapy on their professional and personal lives. Lastly, the psychosocial impact of having cancer at a young age in patients that were previously fit, who may have young families of their own should not be underestimated and the management of this should be incorporated into their routine clinical care. CRC services have traditionally been shaped on the assumption that CRC is a disease of older adults. We believe that both clinical services and research should adapt to take into account the needs of younger patients with CRC.

## 4. Materials and Methods

In this real-world observational study, we included patients with a histologically confirmed diagnosis of CRC between 1 January, 2009 to 31 December, 2014 that were age <50 (range 19–49) at the time of diagnosis. Patients were identified through the use of hospital diagnostic coding. All included patients received systemic anticancer therapy (SACT) at the Royal Marsden Hospital (RMH). Patients that had treatments other than SACT, such as surgery and radiotherapy at other institutions were also included, as long as this information was available for extraction from their electronic patient records.

Data were collected by review of the electronic patient medical records. The demographic data, treatment, response, and survival outcomes were recorded. Radiologic responses were recorded for each treatment line at the first response assessment point, on completion of treatment, and at any subsequent progression assessment point.

All statistical analyses were performed using the STATA 13 software package. The Kaplan–Meier method was used to estimate PFS and OS and the log-rank test was used to compare survival of patient subgroups. A *p*-value < 0.05 was considered to be statistically significant.

This study did not require patient consent to participate. A study protocol outlining the rationale, methods, and statistical analysis plan was approved by an internal institutional review board, the Royal Marsden Hospital’s Committee for Clinical Research, reference number SE474, prior to commencement and is available on request.

## 5. Conclusions

Patients with EOCRC that were treated in a single high-volume UK tertiary center had predominantly left sided, non-hereditary, primaries with advanced stage at diagnosis. Despite treatment with optimal standard of care multimodality therapies, patients with metastatic EOCRC have poorer outcomes than expected. There was an inverse relationship between age and treatment outcomes with patients at the younger end of the metastatic EOCRC spectrum exhibiting inferior mOS.

There is data to suggest that EOCRC is increasing rapidly at a global level. EOCRC is likely to be a molecularly heterogeneous disease that is distinct to that of older patients. New treatment paradigms tailored to target specific EOCRC molecular phenotypes are needed. At the same time clinical services should adapt to take the unique psychosocial needs of patients with EOCRC into account.

## Figures and Tables

**Table 1:** Baseline patient demographics and disease characteristics by stage at diagnosis. ECOG, Eastern Cooperative Oncology Group.

Variable	Stage II *n* (%)	Stage III *n* (%)	Stage IV *n* (%)	All Patients *n* (%)
Stage at diagnosis *	27 (11)	120 (50)	93 (39)	241 (100)
Sex				
Female	13 (48)	47 (39)	46 (49)	107 (44)
Male	14 (52)	73 (61)	47 (51)	134 (56)
Age				
Mean	40	40	41	40
Median	40	43	43	42
Range (min–max)	25–49	25–49	21–49	19–49
<20	0 (0)	1 (<1)	0 (0)	1 (<1)
20–29	2 (7)	12 (10)	10 (11)	24 (10)
30–39	11 (41)	39 (33)	28 (30)	78 (32)
40–49	14 (52)	68 (57)	55 (59)	138 (57)
Ethnic Background				
White British	14 (52)	77 (64)	64 (69)	156 (65)
White Irish	0 (0)	3 (2)	1 (1)	4 (2)
White Other	2 (7)	8 (7)	8 (9)	18 (7)
Asian/Asian-British	2 (7)	14 (12)	8 (9)	24 (10)
African/Caribbean/Black British	4 (15)	10 (8)	4 (4)	18 (7)
Mixed/Multiple (Other Mixed)	1 (4)	0 (0)	0 (0)	1 (<1)
Another Ethnic Group	4 (15)	8 (7)	8 (9)	20 (8)
ECOG PS				
0	12 (44)	24 (20)	16 (17)	52 (22)
1	15 (56)	95 (79)	68 (73)	179 (74)
2	0 (0)	1 (1)	4 (4)	5 (2)
3	0 (0)	0 (0)	5 (5)	5 (2)
Location of primary tumor				
Caecum/ascending colon	5 (18)	31 (26)	21 (23)	57 (24)
Transverse colon	3 (11)	10 (8)	5 (5)	18 (7)
Descending colon/sigmoid	7 (26)	26 (22)	31 (33)	64 (26)
Recto-sigmoid	1 (4)	4 (3)	11 (12)	16 (7)
Rectum	11 (41)	49 (41)	25 (27)	86 (36)
Histology				
Adenocarcinoma	23 (85)	103 (86)	83 (89)	210 (87)
Mucinous adenocarcinoma	4 (15)	16 (13)	3 (3)	23 (10)
Signet cell	0 (0)	1 (1)	5 (5)	6 (2)
Unknown	0 (0)	0 (0)	2 (2)	2 (1)
Primary Tumor differentiation				
Well	1 (4)	3 (2)	0 (0)	4 (2)
Moderate	23 (85)	93 (78)	60 (65)	176 (73)
Poor	3 (11)	21 (18)	20 (21)	45 (19)
Unknown	0 (0)	3 (2)	13 (14)	16 (6)
KRAS				
Mutant	8 (30)	18 (15)	23 (25)	49 (20)
Wild type	4 (15)	35 (29)	46 (50)	85 (35)
Unknown	15 (55)	67 (56)	24 (26)	107 (44)
NRAS				
Mutant	0	0	0	0
Wild type	6 (22)	31 (26)	28 (30)	65 (27)
Unknown	21 (78)	89 (74)	65 (70)	176 (73)
BRAF				
Mutant	0 (0)	8 (7)	4 (4)	12 (5)
Wild type	6 (22)	24 (20)	24 (26)	54 (22)
Unknown	21 (78)	88 (73)	65 (70)	175 (73)
MMR				
Deficient	3 (11)	13 (11)	1 (1)	17 (7)
Proficient	22 (82)	69 (57)	56 (60)	148 (61)
Unknown	2 (7)	38 (32)	36 (39)	76 (32)
Hereditary Syndromes				
Lynch syndrome	3 (11)	7 (6)	1 (1)	11 (5)
FAP	1 (4)	0 (0)	0 (0)	1 (<1)
Other	0 (0)	3 (3)	1 (1)	4 (2)

* 1 patient had stage I rectal cancer at diagnosis.

**Table 2:** Patient outcomes by total number of SACT received in the metastatic setting.

Line of Metastatic SACT	1st	2nd	3rd	4th	5th
Number of Patients	114	78	40	15	6
Best response for each line of SACT *n* (%)	CR 4 (3)	CR 1 (1)	CR 0 (0)	CR 0 (0)	CR 0 (0)
	PR 46 (40)	PR 13 (17)	PR 3 (8)	PR 1 (7)	PR 0 (0)
	SD 26 (23)	SD 20 (26)	SD 9 (23)	SD 2 (13)	SD 2 (33)
	PD 36 (32)	PD 40 (51)	PD 28 (70)	PD 11 (73)	PD 4 (67)
	NA 2 (2)	NA 4 (5)	NA 0 (0)	NA 1 (7)	NA 0 (0)
Regimen modification *n* (%)	43 (38)	17 (22)	4 (10)	3(20)	0
Median PFS for each line of SAC.T (95% CI), months	6.9	5.0	2.0	3.9	2.7
	(5.8–9.4)	(3.4–6.1)	(1.7–3.8)	(1.7–5.4)	(1.5–NA)
Median OS (95% C.I) for patients whose total lines of SACT in metastatic setting was: (months)	9.0	14.9	18.7	31.7	32.0
	(4.1–27.7)	(9.6–19.0)	(14.3–28.6)	(20.246.5)	(N/A)

Legend: SACT: systemic anticancer therapy, CR: complete response, PR: partial response, SD: stable disease, PD: progressive disease, NA: not applicable. PFS: progression-free survival, OS: overall survival.

**Table 3:** Prognostic variables for overall survival in the metastatic setting. mOS, Median overall survival

Prognostic Variable	Number of Patients	mOS (95% C.I) Months	*p*-Value
Histopathology			
Adenocarcinoma	107	20.2 (16.1–26.7)	*p* < 0.001
Mucinous Adenocarcinoma	7	15.8 (NA)	
Signet cells	5	7.0 (NA)	
Location of Primary			
Left	90	22.5 (16.2–27.7)	*p* = 0.18
Right	33	15.9 (9.0–22.0)	
*KRAS* Status *			
Mutant	32	18.5 (11.7–26.2)	*p* = 0.38
Wild Type	56	22.6 (17.1–28.8)	
*BRAF* Status **			
Mutant	7	17.1 (NA)	*p* = 0.45
Wild Type	34	23.2 (16.0–46.5)	

Legend: *p*-value refers to log rank test; * In 35 patient *KRAS* status was unknown; ** In 82 patients *BRAF* status was unknown.
